# The *TCF7L2* rs7903146 (T) allele is associated with type 2 diabetes in urban Ghana: a hospital-based case–control study

**DOI:** 10.1186/1471-2350-14-96

**Published:** 2013-09-23

**Authors:** Ina Danquah, Till Othmer, Laura K Frank, George Bedu-Addo, Matthias B Schulze, Frank P Mockenhaupt

**Affiliations:** 1 Institute of Tropical Medicine and International Health, Charité, Universitätsmedizin Berlin, Spandauer Damm 130, 14050 Berlin, Germany; 2 Department of Molecular Epidemiology, German Institute of Human Nutrition Potsdam-Rehbruecke, Arthur-Scheunert-Allee 114-116, 14558 Nuthetal, Germany; 3 Komfo Anokye Teaching Hospital, School of Medical Sciences, Kwame Nkrumah University of Science and Technology, P.O. Box 1934, Kumasi, Ghana

**Keywords:** Ghana, Type 2 diabetes, TCF7L2, KCNJ11, PPARγ, CAPN10

## Abstract

**Background:**

Type 2 diabetes mellitus is increasing dramatically in sub-Saharan Africa, and genetic predisposition is likely involved in that. Yet, genetic variants known to confer increased susceptibility among Caucasians are far from being established in African populations. In Ghanaian adults, we examined associations of several of these polymorphisms with type 2 diabetes.

**Methods:**

A hospital-based case–control study on type 2 diabetes (and hypertension) was conducted in Kumasi, Ghana. *TCF7L2* rs7903146, *KCNJ11* rs5219, *PPARγ* rs1801282 and *CAPN10* rs3842570, rs3792267, and rs5030952 were typed and associations with type 2 diabetes and phenotypic traits examined.

**Results:**

675 patients with type 2 diabetes and 377 controls were compared. The minor allele frequency of the *TCF7L2* (T) allele was 0.33. In the multivariate model, this allele increased the risk of type 2 diabetes by 39% (95% confidence interval (CI), 1.07-1.81; *p* = 0.014). The minor alleles *KCNJ11* (G) and *PPARγ* (G) were practically absent (each, 0.001). Minor allele frequencies of *CAPN10* were for -43 (A) 0.11 and for -63 (C) 0.46. These variants showed no significant associations with type 2 diabetes. Two *CAPN10* haplotypes tended to protect against type 2 diabetes: 211 (aOR, 0.32; 95% CI, 0.03-1.92; *p* = 0.31) and 221 (aOR, 0.73; 95% CI, 0.48-1.10; *p* = 0.13).

**Conclusions:**

In urban Ghana, the frequency of the *TCF7L2* rs7903146 (T) allele is comparable to the one in Caucasians; the association with type 2 diabetes is slightly weaker. The risk allele *KCNJ11* (G) and the protective allele *PPARγ* (G) are virtually absent. The potential influence of comparatively rare *CAPN10* haplotypes on type 2 diabetes risk in this population requires further evaluation. Large-scale genetic studies among native Africans aiming at fine-mapping the candidate genes are needed to identify the actual factors involved in their increased susceptibility to type 2 diabetes.

## Background

In low-income countries, infectious diseases and malnutrition continue to be the predominant causes of morbidity and mortality [1]. At the same time, these regions are facing an enormous growth of chronic non-communicable diseases, notably type 2 diabetes mellitus. In sub-Saharan Africa (SSA) alone, the number of diabetic patients is projected to double from 15 to 28 million within the next decade [2]. The actual reasons for this development are unclear. Only 3% of published data on type 2 diabetes originate from African populations, mainly black Americans. Overall, Africans are affected earlier by type 2 diabetes and with more severe complications than their Caucasian counterparts. Delayed diagnosis and poor management due to a low socio-economic status (SES) are among the causes. However, differences between blacks and whites in the prevalence and severity of type 2 diabetes do persist even when demographic, socio-economic, behavioral, and clinical parameters are taken into account [3]. This clearly indicates a genetic component in the increased susceptibility of Africans towards this classic polygenic disease [4].

More than 60 susceptibility variants for type 2 diabetes have been identified, and their number increases continuously [5,6]. Among native Africans, the meaning of these polymorphisms for type 2 diabetes is far from being understood. Here, we examined in a Ghanaian population three elsewhere well-established and reproducibly associated type 2 diabetes variants, namely polymorphisms of *transcription factor 7-like 2* (*TCF7L2*), *potassium inwardly-rectifying channel J11* (*KCNJ11*), and *peroxisome proliferator-activated receptor γ* (*PPARγ*) (reviewed by [7]). TCF7L2 is involved in insulin secretion, and the *TCF7L2* rs7903146 single nucleotide polymorphism (SNP) constitutes the best established risk allele in Caucasian populations conferring an overall relative risk for type 2 diabetes of 1.44 [6,8]. The SNP has recently been reported to be strongly associated with type 2 diabetes also in African Americans (adjusted odds ratio (OR), 1.37) [8]. Notably, in native Africans, the role of *TCF7L2* rs7903146 has been examined only once, however, in an ethnically mixed population of Ghanaians and Nigerians, yielding an OR of 1.45 [9].

Two further classic type 2 diabetes risk alleles, *KCNJ11* rs5219 (G) (influencing insulin secretion) and *PPARγ* rs1801282 (C) (influencing insulin sensitivity) are considered to be very rare and very frequent, respectively, in individuals of African descent [8,10-12]. Irrespective of the consequently limited power of detecting associations with type 2 diabetes, we aimed at confirming their frequencies in a native West African population.

Lastly, we examined *CAPN10* variants (rs3792267 (-43), rs3842570 (-19), and rs5030952 (-63) [4]) of the Ca^2+^-dependent cysteine protease calpain 10 which may influence both, insulin secretion and peripheral sensitivity. In the only study among native West Africans, *CAPN10* polymorphisms and type 2 diabetes were not associated, even though the potential risk allele -43 (A) was more frequent among patients with type 2 diabetes [13]. Notably, -43 (G) is considered the type 2 diabetes risk allele in some studies [4], while it confers improved insulin secretion in others [14,15].

The aim of the present study was therefore to examine the allele frequencies of selected genetic polymorphisms, common and replicated in other populations, among urban Ghanaians as well as the associations with type 2 diabetes and phenotypic traits.

## Methods

### Study site and design

The study was conducted from August 2007 through June 2008 at Komfo Anokye Teaching Hospital (KATH), Kumasi, Ghana. Detailed descriptions of the study site and the recruitment procedures are presented elsewhere [16]. In brief, about 6% of the adult population in this region exhibit type 2 diabetes, 29% have hypertension, and roughly 23% are overweight [17-19]. The diabetes center and the hypertension clinic at the hospital each deliver health care to >100 patients per week, the majority of whom are urban residents living on small-scale trading. The unmatched case–control study was designed to primarily identify factors associated with type 2 diabetes. The present analysis aims at genetic associations with type 2 diabetes and with diabetic traits among the control group.

The study protocol was reviewed and approved by the Ethics Committee, School of Medical Sciences, University of Science and Technology, Kumasi, and informed written consent was obtained from all participants.

### Recruitment procedures and examinations

After providing detailed information on the objectives and procedures of the study, patients were recruited from the diabetes center (n = 495) and the hypertension clinic (n = 451). Patients encouraged members of their community (n = 222) to participate in the study as preliminary controls. Likewise, further preliminary controls were recruited among outpatients (n = 150) and hospital staff (n = 148). After exclusion of type 2 diabetes and hypertension (see below), they were included into the study as controls.

All participants were instructed on fasting, abstaining from cigarette smoking, alcohol and coffee consumption as well as on avoiding excessive physical activity beginning at 10:00 p.m. the day before examination. On the day of examination, each participant underwent a measurement of fasting plasma glucose (FPG). The socio-demographic data were documented; the participants were physically examined and interviewed with respect to residence, own and family history of diabetes and hypertension, medications, smoking behavior and education. Venous blood and urine samples were collected. Weight, height, waist and hip circumferences were measured (all devices Seca, Germany). After 10 minutes resting time in an air-conditioned examination room, blood pressure and heart rate were assessed at 0, 5, and 10 min. (M8 Comfort, Omron, Japan).

### Laboratory analyses

FPG (fluoride whole blood, tubes cooled at +4°C) and urinary albumin were measured photometrically (Glucose 201+ & Albumin Systems; HemoCue, Ångelholm, Sweden). Glucose concentration is presented as plasma equivalents. The coefficients of variation ranged between 1.7-6.1% and 4.9-8.0%, respectively.

DNA was extracted from stabilized full blood aliquots (stabilizing buffer AS1 and QIAamp DNA blood mini kit, Qiagen, Germany) [20]. *TCF7L2* rs7903146, *KCNJ11* rs5219 and *PPARγ* rs1801282 were genotyped by the LightCycler 480 device (Roche Diagnostics, Mannheim, Germany) using commercially available primers and probes (TIB MOLBIOL, Berlin, Germany) (Additional file 1: Table S1). *CAPN10* rs3792267 was detected by a mutagenically separated PCR method, which uses a common forward primer and two allele-specific reverse primers. As for *CAPN10* rs3842570, this insertion/deletion polymorphism was amplified by forward and reverse primers, whereas *CAPN10* rs5030952 was identified as restriction fragment length polymorphism [21]. For each batch of genotyping assay, positive controls of known sequence were included and the evaluation of results was done manually as well as per individual sample. Whenever an assay was negative, unclear or implausible, it was repeated.

### Power analysis

For the most prominent candidate variant of type 2 diabetes susceptibility, i.e., *TCF7L2* rs7903146, we assumed an additive effect, yielding an OR of at least 1.35 [9]. At an allele frequency of 0.30, given a significance level of α = 0.05 and a 95% confidence interval (CI), the present sample size of 1052 (2 cases vs. 1 control) is sufficient to replicate this OR with a probability (power) of 84%. Given minor allele frequencies (MAFs) of 0.11, 0.23 and 0.46 for *CAPN10* –43, –19 and -63 at an expected diabetes prevalence of 6%, an OR of 1.30 can be detected at the given sample size (675 cases and 377 controls) with a power of 66%, 67% and 79%, respectively. Power calculations have been performed using Quanto 1.2.4 [22].

### Data management and analyses

Body mass index (BMI) was calculated as weight in kg divided by height squared in m. Overweight and obesity were defined as BMI ≥25.0-29.9 kg/m^2^ and ≥30.0 kg/m^2^, respectively. Central adiposity was defined as waist circumference ≥102 cm (male) and ≥88 cm (female). Type 2 diabetes was defined as FPG ≥7 mmol/l and/or documented anti-diabetic medication. Hypertension denoted a mean of blood pressure ≥140/90 mmHg of three independent measurements and/or documented anti-hypertensive treatment. Controls were negative for both conditions. Microalbuminuria was defined as ≥20 mg/l.

Distributions of categorical and continuous parameters were compared between diabetic patients and controls by χ^2^-test and Mann–Whitney-U-test, respectively. ORs for type 2 diabetes, 95% CI and *p* values were calculated by logistic regression analyses. *CAPN10* haplotypes were reconstructed from genotype data using the software package PHASE version 2.1 [23,24]. With respect to genetic polymorphisms, we assumed an additive effect of candidate alleles on type 2 diabetes risk (homozygous for non-risk allele, 0; heterozygous for risk allele, 1; homozygous for risk allele, 2). The crude odds ratio (OR) and a multivariate estimate adjusted for age, gender, BMI ≥25.0 kg/m^2^ and hypertension status were calculated. In a second analysis, associations of SNPs with phenotypic traits among the controls were calculated using linear regression models. These traits were waist circumference, BMI, FPG, systolic and diastolic blood pressure, and urinary albumin. Non-normally distributed parameters were log_e_ transformed.

## Results

### Study population

Overall, we recruited 1466 study participants, of whom 46% (675) presented with type 2 diabetes and 26% (377) were controls; further 414 (28%) had hypertension only and were not regarded for the present analysis. The detailed description of the study population including demographic characteristics, clinical and anthropometric data, medical history, physical activity, nutritional behavior and socioeconomic background is presented elsewhere [16].

The majority of patients with type 2 diabetes (97%) regularly attended the diabetes center, presenting with a median duration of type 2 diabetes of 5.0 (interquartile range, 2.0-9.0) years. Characteristics of patients and controls are presented in Table 1. The participants were mainly female, middle-aged, overweight and of low SES. As compared to controls, diabetic patients were significantly older (*p* < 0.001), more often centrally adipose (69% vs. 53%; *p* < 0.001) and had the lowest SES (unemployment, 37% vs. 10%, *p* < 0.001). Hypertension (63%) and microalbuminuria (43%) were frequent among diabetic patients. Also, they more often reported a family history of diabetes and hypertension than controls (each *p* < 0.001). Metformin-based therapies (78%) and combinations with sulfonylureas (61%) were predominating [16]. Except for increased rates of smoking among type 2 diabetes cases, no further differences were observed compared to controls. Self-reported Akan ethnicity was 88% in the type 2 diabetes group and 84% among controls (*p* = 0.11).

**Table 1:** Characteristics of 1052 urban Ghanaians

**Characteristics**	**Controls**	**Type 2 diabetes**	***P***
*N*	377	675	
Age (years)	38.8 ± 14.8	54.7 ± 13.4	<0.001
Sex (female)	76.4 (288)	74.7 (504)	0.50
Residence (Kumasi metropolitan area)	79.3 (299)	70.8 (476)	0.003
Ethnic group (Akan)	84.4 (318)	87.7 (592)	0.11
Systolic blood pressure (mmHg)	116.0 ± 11.3	138.3 ± 23.9	<0.001
Diastolic blood pressure (mmHg)	76.0 ± 7.5	85.1 ± 12.1	<0.001
Urinary albumin (mg/l)	9.0 (4.9–150.1)	14.5 (3.3–150.1)	<0.001
Waist circumference (cm)	81.5 ± 12.1	91.1 ± 12.2	<0.001
Body mass index (kg/m^2^)	24.6 ± 4.9	25.9 ± 5.1	<0.001
Diabetes family history (yes)	26.3 (99)	57.9 (391)	<0.001
Hypertension family history (yes)	30.2 (114)	40.7 (275)	<0.001
Smoking status (ever) ^a^	3.7 (14)	7.3 (49)	0.02
Formal education (none)	10.9 (41)	35.7 (240)	<0.001
Occupation			
Public servant	27.4 (103)	6.5 (44)	
Trader	25.0 (94)	29.5 (198)	
Farmer	3.2 (12)	9.7 (65)	
Else	34.6 (130)	17.4 (117)	
Unemployed	9.8 (37)	36.9 (248)	<0.001

Values are expressed as means ± standard deviation, median (range) or % (*n*). ^a^, include current and quit smoking.

### Genetic variants and type 2 diabetes

Genotyping success was 97.1% for *PPARγ* rs1801282, 99.6% for each variant of *CAPN10* (rs3842570, rs3792267, rs5030952), 99.7% for *TCF7L2* rs7903146 and 100% for *KCNJ11* rs5219. All genotypes obeyed the Hardy-Weinberg equilibrium. Minor allele frequencies and associations with type 2 diabetes are presented in Table 2.

**Table 2:** Associations of common variants with type 2 diabetes in urban Ghana

**Gene SNP**	**Genotype**	**N**	**Controls**	**Type 2 diabetes**	**OR (9% CI)**	***P***	**aOR (95% CI)**^**a**^	***P***
*TCF7L2* rs7903146	n	1049	375	674				
	C/C	455	182 (48.5)	273 (40.5)	Reference			
	C/T	488	165 (44.0)	323 (47.9)				
	T/T	106	28 (7.5)	78 (11.6)	1.34 (1.10–1.63)	0.004	1.39 (1.07–1.81)	0.014
	MAF (T)		0.30	0.36				
*KCNJ11* rs5219	n	1052	377	675				
	A/A	1051	377 (100)	674 (99.9)	Reference			
	A/G	1	0 (0)	1 (0.1)				
	G/G	0	0 (0)	0 (0)	-		-	
	MAF (G)		0.0	0.0007				
*PPARγ* rs1801282	n	1021	365	656				
	C/C	1020	365 (100)	655 (99.8)	Reference			
	C/G	1	0 (0)	1 (0.2)				
	G/G	0	0 (0)	0 (0)	-		-	
	MAF (G)		0.0	0.0008				
*CAPN10* rs3792267	n	1048	375	673				
	G/G	826	289 (77.1)	537 (79.8)	Reference			
	G/A	209	80 (21.3)	129 (19.2)				
	A/A	13	6 (1.6)	7 (1.0)	0.85 (0.64–1.12)	0.25	0.70 (0.47–1.03)	0.07
	MAF (A)		0.13	0.11				
*CAPN10* rs3842570	n	1048	374	674				
	2 repeats/2 repeats	622	225 (60.2)	397 (58.9)	Reference			
	2 repeats/3 repeats	376	128 (34.2)	248 (36.8)				
	3 repeats/3 repeats	50	21 (5.6)	29 (4.3)	1.00 (0.80–1.24)	0.99	1.12 (0.84–1.49)	0.45
	MAF (3 repeats)		0.23	0.23				
*CAPN10* rs5030952	n	1048	374	674				
	C/C	219	79 (21.1)	140 (20.8)	Reference			
	C/T	523	182 (48.7)	341 (50.6)				
	T/T	306	113 (30.2)	193 (28.6)	0.98 (0.81–1.17)	0.79	0.98 (0.76–1.25)	0.84
	MAF (T)		0.55	0.54				

MAF, minor allele frequency. ^a^, The multivariate odds ratio is adjusted for age, gender, BMI ≥25.0 kg/m^2^ and hypertension status.

The *TCF7L2* (T) allele was increased in the type 2 diabetes group (0.36) compared to the controls (0.30; *p* = 0.01). In multivariate analysis adjusting for age, gender, BMI ≥25.0 kg/m^2^ and hypertension status, *TCF7L2* (T) was associated with increased odds of type 2 diabetes (aOR, 1.39; Bonferroni-corrected *p* = 0.056). Due to the low MAFs of *KCNJ11* and *PPARγ* (Table 2), the calculation of risk estimates did not yield meaningful results. The MAFs of *CAPN10* variants did not differ between diabetic patients and controls. Constructing haplotypes, the most frequent combination of alleles -43 (A), –19 (3 repeats), and -63 (T) was 112 at 0.53, followed by 111 (0.24), 121 (0.12), 221 (0.10), 222 (0.01), 122 (0.004), and 211 (0.003). None of these conferred a significant risk for type 2 diabetes. In contrast, two haplotypes were nominally associated with reduced odds for type 2 diabetes: 211 (aOR, 0.32; 95% CI, 0.03-2.92; *p* = 0.31) and 221 (aOR, 0.73; 95% CI, 0.48-1.10; *p* = 0.13) (Table 3).

**Table 3:** **Associations of *CAPN10 *haplotypes with type 2 diabetes in urban Ghana**

**Haplotype**^**a**^	**Genotype**	**N**	**Controls**	**Type 2 diabetes**	**OR (95% CI)**	***P***	**aOR (95% CI)**^**b**^	***P***
*111*	n	1048	374	674				
	- -	608	219 (58.6)	389 (57.7)	Reference			
	111 -	376	140 (37.4)	236 (35.0)				
	111 111	64	15 (4.0)	49 (7.3)	1.12 (0.91–1.38)	0.30	1.00 (0.75–1.34)	0.98
	Frequency		0.23	0.25				
*112*	n	1048	374	674				
	- -	224	82 (21.9)	142 (21.1)	Reference			
	112 -	544	183 (48.9)	361 (53.6)				
	112 112	280	109 (29.1)	171 (25.4)	0.94 (0.78–1.13)	0.51	0.95 (0.74–1.22)	0.67
	Frequency		0.54	0.52				
*121*	n	1048	374	674				
	- -	823	296 (79.1)	527 (78.2)	Reference			
	121 -	205	72 (19.3)	133 (19.7)				
	121 121	20	6 (1.6)	14 (2.1)	1.07 (0.81–1.40)	0.64	1.40 (0.99–1.99)	0.06
	Frequency		0.11	0.12				
*122*	n	1048	374	674				
	- -	1039	372 (99.5)	667 (99.0)	Reference			
	122 -	9	2 (0.5)	7 (1.0)				
	122 122	0	0 (0.0)	0 (0.0)	1.95 (0.40–9.44)	0.41	3.87 (0.61–24.52)	0.15
	Frequency		0.01	0.01				
*221*	n	1048	374	674				
	- -	853	298 (79.7)	555 (82.3)	Reference			
	221 -	186	72 (19.3)	114 (16.9)				
	221 221	9	4 (1.1)	5 (0.7)	0.84 (0.63–1.14)	0.27	0.73 (0.48–1.10)	0.13
	Frequency		0.11	0.09				
*211*	n	1048	374	674				
	- -	1041	369 (98.7)	672 (99.7)	Reference			
	211 -	7	5 (1.3)	2 (0.3)				
	211 211	0	0 (0.0)	0 (0.0)	0.22 (0.04–1.14)	0.07	0.32 (0.03–2.92)	0.31
	Frequency		0.01	0.001				
*212*	n	1048	374	674				
	- -	1043	372 (99.5)	671 (99.6)	Reference			
	212 -	5	2 (0.5)	3 (0.4)				
	212 212	0	0 (0.0)	0 (0.0)	0.83 (0.14–5.00)	0.84	-	/
	Frequency		0.003	0.002				
*222*	N	1048	374	674				
	- -	1030	370 (98.9)	660 (97.9)	Reference			
	222 -	18	4 (1.2)	14 (2.1)				
	222 222	0	0 (0.0)	0 (0.0)	1.96 (0.64–6.00)	0.24	1.20 (0.28–5.04)	0.81
	Frequency		0.01	0.01				

^a^, Haplotypes were constructed from genotype data using the software package PHASE version 2.1 [23,24].

^b^, Adjusted for age, gender, BMI ≥25.0 kg/m^2^ and hypertension status.

### Genetic variants and diabetic traits

In linear regression models, we investigated whether *TCF7L2* and *CAPN10* variants were associated with phenotypic characteristics among controls without type 2 diabetes, including log_e_-normalized FPG, waist circumference, BMI, systolic and diastolic blood pressure and urinary albumin. For *TCF7L2* rs7903146, median FPG (IQR) significantly increased with the number of T alleles: C/C, 4.4 (4.0-4.7); C/T, 4.5 (4.1-5.0); T/T, 4.7 (4.4-5.4) mmol/L (*p* = 0.001). This association remained after adjustment for age, gender, BMI and hypertension status (*p* = 0.001). Also, a non-significant tendency for increased diastolic blood pressure was observed in participants carrying both risk alleles: C/C, 76 (70–81); C/T, 76 (71–81); T/T, 81 (75–84) mmol/L (*p* = 0.1). Body composition and urinary albumin concentrations were not influenced by *TCF7L2* rs7903146 (all *p* >0.3). As for the *CAPN10* variants, no associations with phenotypic characteristics in the control group were observed (data not shown).

## Discussion

The importance of genetic variants for type 2 diabetes established in western populations is far from being understood for sub-Saharan Africans. Here, we have investigated the role of three type 2 diabetes candidate SNPs well-known in other populations (*TCF7L2* rs7903146, *KCNJ11* rs5219, *PPARγ* rs1801282) in more than 1000 Ghanaians. Because of ambiguous findings in another West African study [13], we also included *CAPN10* variants. More than half of the individuals carried the *TCF7L2* (T) allele, which was suggestive to increase the odds for type 2 diabetes by roughly 40%. Also, in healthy controls, FPG was significantly elevated in carriers of this allele. Remarkably, the *KCNJ11* (G) and *PPARγ* (C) alleles were practically absent. The respective minor alleles of *CAPN10* variants were frequent but not associated with type 2 diabetes.

These findings need to be interpreted with caution. One study limitation is the comparatively small sample size that did not allow detecting effects of rare variants, such as of *PPARγ* and *KCNJ11*, and which may also contribute to the possibility of type I statistical errors. Clearly, larger studies purposely designed for determining the relevance of specific SNPs on a population-wide level are required. Also, extensive genetic admixture within the Ghanaian population may have obscured our findings. Ancient and recent migration from neighbouring countries and North Africa seems responsible for a highly variable genetic structure [25]. Even though, the majority of our participants claimed to be of Akan ethnicity, and this was equally true for controls and cases with type 2 diabetes, we cannot exclude admixture from other ethnic groups. As a potential drawback, genotyping in duplicates was not done systematically. Nevertheless, all genotypes were in Hardy-Weinberg equilibrium, arguing against major typing errors. Study participants recruited in hospital may not reflect the genetic make-up of the average Ghanaian population, particularly when the ratio of cases and controls is 2:1. On the other hand, neither improved awareness of type 2 diabetes among patients nor management will influence predisposition and will therefore not affect associations of the genetic variants with type 2 diabetes. A major limitation lies in the unmatched design of our case–control study. Controls were younger, leaner, and had less hypertension as well as a higher SES than patients. Some controls may consequently show increased FBG and possibly diabetes when they become older and/or gain weight. In multivariate analysis, we have accounted for the differences in age (and gender, obesity, and hypertension) between cases and controls. Nevertheless, we cannot rule out residual attenuation by an over-representation of young participants in the control group. We are aware that the definition of type 2 diabetes by single FPG measurement and known medications is sub-optimal. However, it corresponds to general practice in resource-poor settings and IDF consensus [26]. Glycated haemoglobin was not used for the diagnosis of type 2 diabetes as the high prevalences of hemoglobinopathies and haemolytic conditions, such as malaria, may have complicated the interpretation [27].

The present study provides first-time insight into the role of common polymorphisms – previously associated with type 2 diabetes in other populations – among a comparatively large and presumably non-admixed population of SSA. So far, most of the work in African populations, mainly African Americans, has focused on the *TCF7L2* variant, revealing MAFs of around 0.34 and ORs for type 2 diabetes of 1.37 [8,9,11]. In Caucasians, these figures are 0.28-0.32 and 1.44 [8]. Two small African studies, report MAFs of 0.26-0.48 and diverse risk estimates (ORs: Nigeria, 1.7; Ghana, 1.0, South Africa, 1.3) [9,11]. We confirm the high frequency of the T allele in West-Africa as well as its association with type 2 diabetes and increased FPG. Indeed, these observations were independent of BMI status, supporting the concept of reduced insulin secretion *via* a deficiency of the gene product TCF7L2 [4]. Interestingly, previous studies have revealed that frequencies of the T allele are lowest in North-America and Europe (with a gradient of increasing frequency from North to South), moderate in Asia, and highest in Africa [8,9,28].

Surprisingly, we are the first to investigate the importance of *PPARγ* rs1801282 and *KCNJ11* rs5219 for type 2 diabetes in West Africa. Almost everybody in the present study population displayed the risk allele of *PPARγ* (C) while the risk allele of *KCNJ11* (G) was almost absent. The protective *PPARγ* (G) variant has a global allele frequency of around 0.10, with highest figures in northern Europeans [29]. In Caucasian and Asian populations, the G variant commonly protects against type 2 diabetes (OR Caucasians, 0.9; OR Asians, 0.8) [8,10], particularly in populations with a high lipid contribution to energy intake [30]. For SSA, associations with type 2 diabetes are conflicting [8,10].

As for *KCNJ11* rs5219, only few studies have examined the importance of the G allele in blacks: In an African American population, it was associated with reduced odds of type 2 diabetes; the frequency was 0.06 [12]. A recent meta-analysis of eight African American cohort studies has found an increased risk for type 2 diabetes by 10% [8]; and the G allele was absent in a South African population of Zulu descent [11]. Findings are inconsistent in Asian populations, while the variant is a robust marker for type 2 diabetes in Caucasians [8,31]. The functional role of the SNP remains unclear. It is known to promote hypoinsulinemia, reduction of body weight and physical endurance [32]. However, activation of the gene product depends on several other co-factors that may outweigh the importance of the SNP [33,34]. The near absence of the G allele in our study population and its contradictory influence in other regions argue for alternative variants influencing the population-wide variation in type 2 diabetes risk in SSA.

*CAPN10* polymorphisms are associated with diabetic status in Mexican Americans (OR, 2.8), Botnian Fins (OR, 2.5) and Germans (OR, 5.0) [35]. Subsequent studies, however, could not replicate the strength of association in Caucasians [36], and the role in African populations is unclear [12,13,37]. Here, we replicated the allele frequencies of the most prominent *CAPN10* variants in West Africans. These were neither associated with type 2 diabetes nor with diabetic traits in this urban Ghanaian population. The co-existence of susceptibility variants and protective haplotypes, previously reported from Caucasian and Indian populations [36,38], may be responsible for these findings. Indeed, some *CAPN10* haplotypes nominally confer a protection against type 2 diabetes in our study population (ORs, 0.45-0.72). However, the figures contrast previous findings from two ethnic groups in Ghana, where the 221 haplotype showed no association with type 2 diabetes (OR, 0.9) [13]. Clearly, further investigations are warranted to understand the role of *CAPN10* variants and their interplay for the risk of type 2 diabetes in SSA.

## Conclusions

In conclusion, the *TCF7L2* rs7903146 (T) allele, the most unequivocal genetic factor influencing type 2 diabetes among Caucasians, is very common among Ghanaians and associated with type 2 diabetes and FPG. The degree of association is slightly weaker than the one in Europe. The protective allele of *PPARγ* rs1801282 and the risk-conferring allele of *KCNJ11* rs5219 are nearly absent and thus have a debatable relevance for population-wide variation in type 2 diabetes risk in this area. *CAPN10* polymorphisms in SSA might influence type 2 diabetes risk only in certain combinations. These results demonstrate the need for the identification of ethnicity-specific genetic associations with type 2 diabetes in SSA. The present replications of single but well-established loci from other populations can only serve as the first step to fine-map the candidate genes and explore their specific associations with type 2 diabetes in this region.

## Abbreviations

CAPN10: Calpain 10; CI: Confidence interval; FPG: Fasting plasma glucose; IDF: International Diabetes Federation; KATH: Komfo Anokye Teaching Hospital; KCNJ11: Potassium inwardly-rectifying channel J11; OR: Odds ratio; PPARγ: Peroxisome proliferator-activated receptor gamma; SES: Socio-economic status; SNP: Single nucleotide polymorphism; SSA: Sub-Saharan Africa; TCF7L2: Transcription factor 7 like 2.

## Competing interests

The authors declared that they have no competing interests.

## Authors’ contributions

ID was responsible for study conception, on-site recruitment, data analysis and manuscript writing; TO did the genotyping and reviewed the manuscript; LKF contributed to manuscript writing and did the technical review; GB was responsible for on-site recruitment including data collection and contributed to data interpretation and manuscript writing; MBS contributed to data interpretation and reviewed the manuscript; FPM is the guarantor of the manuscript, he was involved in study design, coordinated the recruitment and contributed to manuscript writing. All authors read and approved the final manuscript.

## Pre-publication history

The pre-publication history for this paper can be accessed here:

http://www.biomedcentral.com/1471-2350/14/96/prepub

## Supplementary Material

Additional file 1: Table S1 Genotyping protocols for TCF7L2 rs7903146, KCNJ11 rs5219 and PPAR rs1801282. Click here for file
