# Supplementary material for: The TCF7L2 rs7903146 (T) allele is associated with type 2 diabetes in urban Ghana: a hospital-based case–control study
Source: BMC Med Genet. 2013 Sep 23;14:96. doi: 10.1186/1471-2350-14-96 (PMC3848778; doi:10.1186/1471-2350-14-96)
Supplement: Additional file 1: Table S1 — Genotyping protocols for TCF7L2 rs7903146, KCNJ11 rs5219 and PPAR rs1801282. [file 1471-2350-14-96-S1.pdf]

Supplementary Table 1 Genotyping protocols for TCF7L2 rs7903146, KCNJ11 rs5219 and PPARγ rs1801282

SOP LightCycler LC480

Method		TCF7L2 rs7903146 [TCF4]			TIB-Produkt Nr.
**Primer**					
forward	**TCF4 S**	GGT GAC AAA TTC ATG GGC TTT CT			779752
reverse	**TCF4 A**	CGT CTG AAA ACT AAG GGT GAC TCA TA			779753
**Probes**					
Sensor	**Sensor C**	LC640-CTT TTT AGA TAC TAT ATA ATT TAA TTG CCG-PH			779757
Anchor	**Anchor TCF4**	GCT GTT ATT TAC TGA ACA ATT AGA GAG CTA AGC-FL			779754
**PCR Mix**					
**Component**	**final Conc.**	**1x**	**100x**		
5x Genotyping Master (Roche)	0,5x	1 µl	100 µl		
$MgCl_2$ (25mM)	3 mM	0,6 µl	60 µl		
Primer f (10µM)	1,25 pmol	0,125 µl	12,5 µl		
Primer A (10µM)	5 pmol	0,5 µl	50 µl		
Sensor (3µM)	1,5 pmol	0,5 µl	50 µl		
Anchor (3µM)	1,5 pmol	0,5 µl	50 µl		
H_2O		3,225 µl	322,5 µl		
Template DNA	~ 30 ng/µl	1 µl			
**total volume/well**		**10 µl**			
**Setup**					
**Detection Format**	**Block Type**		**Reaction Volume**		
Mono Color HybProbe	96		10		
**Program**					
**Program Name**	**Cycles**		**Analysis Mode**		
Pre-Incubation	1		None		
Amplification	40		Quantification		
Melting Curve	1		Melting Curves		
Cooling	1		None		
**Temperature Targets**					
**Target (^{0}C)**	**Acquisition Mode**	**Hold (hh:mm:ss)**	**Ramp Rate (^{0}C/s)**	**Acquisitions (per ^{o}C)**	
**Pre-Incubation**					
95	None	00:10:00	4.4	-	
**Amplification**					
95	None	00:00:05	4.4	-	40x
60	Single	00:00:20	1.5	-	
72	None	00:00:10	4.4	-	
**Melting Curve**					
95	None	00:00:30	4.4	-	
60	None	00:00:30	1.5	-	
53	None	00:00:30	1.5	-	
45	None	00:01:00	1.5	-	
70	Continuous	-	-	3	
**Cooling**					
40	None	00:00:30	2.2	-	

SOP LightCycler LC480

**Method**		**KCNJ11 rs5219**			TIB-Produkt Nr.
**Primer**					
forward	**KCNJ f**	GGG GTC ACC GGA GCC AT			1141215
reverse	**KCNJ A**	TGT GGG CCA CGT TGC A			1141217
**Probes**					
Sensor	**Sensor [A]**	AGG ACC CTG CCA AGC CCA—FL			1141219
Anchor	**Anchor**	LC640—TAC CGT GCC CGC CAG CGG AG—PH			1141220

PCR Mix

**Component**	**final Conc.**	**1x**	**100x**		
5x Genotyping Master (Roche)	0,5x	1 µl	100 µl		
$MgCl_2$ (25mM)	3 mM	0,6 µl	60 µl		
Primer f (10µM)	1,25 pmol	0,125 µl	12,5 µl		
Primer A (10µM)	5 pmol	0,5 µl	50 µl		
Sensor (3µM)	1,5 pmol	0,5 µl	50 µl		
Anchor (3µM)	1,5 pmol	0,5 µl	50 µl		
H_2O		3,225 µl	322,5 µl		
Template DNA	~ 30 ng/µl	1 µl			
**total volume/well**		**10 µl**			

Setup

**Detection Format**	**Block Type**	**Reaction Volume**
Mono Color HybProbe	96	10

Program

**Program Name**	**Cycles**	**Analysis Mode**
Pre-Incubation	1	None
Amplification	40	Quantification
Melting Curve	1	Melting Curves
Cooling	1	None

Temperature Targets

**Target (^{0}C)**	**Acquisition Mode**	**Hold (hh:mm:ss)**	**Ramp Rate (^{0}C/s)**	**Acquisitions (per ^{0}C)**	
**Pre-Incubation**					
95	None	00:10:00	4.4	-	
**Amplification**					
95	None	00:00:05	4.4	-	
58	Single	00:00:10	1.5	-	40x
72	None	00:00:15	4.4	-	
**Melting Curve**					
95	None	00:00:30	4.4	-	
66	None	00:00:30	1.5	-	
57	None	00:00:30	1.5	-	
45	None	00:01:00	1.5	-	
75	Continuous	-	-	3	
**Cooling**					
40	None	00:00:30	2.2	-	

SOP LightCycler LC480

Method		hu PPARG rs1801282			TIB-Produkt Nr.
**Primer**					
forward	**PPARGex1 S**	ATT TGG AAA CTG ATG TCT TGA CTC A			779750
reverse	**PPARGin1 A**	CAA TAG CCG TAT CTG GAA GGA AC			779751
**Probes**					
Sensor	**Sensor [G]**	CTC CTA TTG ACG CAG AAA GCG-FL			779755
Anchor	**PPARG Anc**	LC640-TCC TTC ACT GAT ACA CTG TCT GCA AAC ATA TC-PH			779756
**PCR Mix**					
**Component**	**final Conc.**	**1x**	**100x**		
5x Genotyping Master (Roche)	0,5x	1 µl	100 µl		
$MgCl_2$ (25mM)	3 mM	0,6 µl	60 µl		
Primer S (10µM)	1,25 pmol	0,125 µl	12,5 µl		
Primer A (10µM)	5 pmol	0,5 µl	50 µl		
Sensor (3µM)	1,5 pmol	0,5 µl	50 µl		
Anchor (3µM)	1,5 pmol	0,5 µl	50 µl		
H_2O		3,225 µl	322,5 µl		
Template DNA	~ 30 ng/µl	1 µl			
**total volume/well**		**10 µl**			
**Setup**					
**Detection Format**	**Block Type**		**Reaction Volume**		
Mono Color HybProbe	96		10		
**Program**					
**Program Name**	**Cycles**		**Analysis Mode**		
Pre-Incubation	1		None		
Amplification	40		Quantification		
Melting Curve	1		Melting Curves		
Cooling	1		None		
**Temperature Targets**					
**Target (^{0}C)**	**Acquisition Mode**	**Hold (hh:mm:ss)**	**Ramp Rate (^{0}C/s)**	**Acquisitions (per ^{o}C)**	
**Pre-Incubation**					
95	None	00:10:00	4.4	-	
**Amplification**					
95	None	00:00:05	4.4	-	40x
58	Single	00:00:15	1.5	-	
72	None	00:00:12	4.4	-	
**Melting Curve**					
95	None	00:00:30	4.4	-	
62	None	00:00:30	1.5	-	
54	None	00:00:30	1.5	-	
48	None	00:01:00	1.5	-	
75	Continuous	-	-	3	
**Cooling**					
40	None	00:00:30	2.2	-	
